# Embryonic senescent cells re-enter cell cycle and contribute to tissues after birth

**DOI:** 10.1038/s41422-018-0050-6

**Published:** 2018-06-05

**Authors:** Yi Li, Huan Zhao, Xiuzhen Huang, Juan Tang, Shaohua Zhang, Yan Li, Xiuxiu Liu, Lingjuan He, Zhengyu Ju, Kathy O. Lui, Bin Zhou

**Affiliations:** 1 State Key Laboratory of Cell Biology, CAS Center for Excellence in Molecular Cell Science, Institute of Biochemistry and Cell Biology, Shanghai Institutes for Biological Sciences, University of Chinese Academy of Sciences, Chinese Academy of Sciences, Shanghai, 200031 China; 2 Key Laboratory of Nutrition and Metabolism, Institute for Nutritional Sciences, Shanghai Institutes for Biological Sciences, University of Chinese Academy of Sciences, Chinese Academy of Sciences, Shanghai, 200031 China; 3 Key Laboratory of Regenerative Medicine of Ministry of Education, Institute of Aging and Regenerative Medicine, Jinan University, Guangzhou, Guangdong 510632 China; 4 Department of Chemical Pathology, Li Ka Shing Institute of Health Sciences, The Chinese University of Hong Kong, Prince of Wales Hospital, Shatin, Hong Kong SAR China; 5 School of Life Science and Technology, ShanghaiTech University, Shanghai, 201210 China

Dear Editor,

Cellular senescence (or senescence) has been regarded as a stable form of cell cycle arrest by in vitro cell culture experiments.^1^ Recent studies indicate that senescence is associated with aging and diseases, including cancers.^2,3^ For instances, it suppresses tumor progression by halting the growth of premalignant cells,^4^ and promotes wound healing by preventing excessive tissue fibrosis or induction of cell dedifferentiation.^5,6^ Targeting senescent cells could restore tissue homeostasis in response to aging, chemotoxicity, or injury.^7^ In addition to these pathological conditions in adults, cellular senescence also occurs in physiological states such as mammalian mouse^8,9^ and human^10,11^ embryonic development. Embryonic senescent cells have been reported to be non-proliferative and subjected to clearance from tissues after apoptosis at late embryonic stage.^8,9^ However, the interpretation for clearance of senescent cells at late embryonic stage is based on the disappearance of Cdkn1a (P21) expression and senescence-associated beta-galactosidase (SAβ-Gal) activity,^8,9^ two commonly used senescence markers in the field. Currently, there is no genetic fate mapping evidence for senescent cell fate in vivo. By lineage tracing of P21^+^ senescent cells, we found that embryonic senescent cells labeled at mid-embryonic stage gradually lost P21 expression and SAβ-Gal activity at late embryonic stage. Unexpectedly, some of the previously labeled senescent cells re-entered the cell cycle and proliferated in situ. Moreover, these previously labeled senescent cells were not cleared at late embryonic stage and remained in the tissue after birth. This study unravels in vivo senescent cell fates during embryogenesis, indicating their potential plasticity.

We first performed SAβ-Gal staining on embryos and found SAβ-Gal^+^ signals in the apical ectodermal ridge (AER) at E10.5–E14.5. We hardly detected positive signals in the AER at E15.5 and afterwards (Fig. 1a). SAβ-Gal activity in AER was validated by staining on tissue sections (Supplementary information, Figure S1a). To confirm the specificity of SAβ-Gal staining for senescence (pH 6.0), we stained embryos at pH 6.5 and pH 7.0 for technical controls as previously described.^8^ Indeed, we did not detect any positive SAβ-Gal signal at E10.5–E14.5 (Supplementary information, Figure S1b). These results were consistent with previous studies,^8,9^ demonstrating that senescent cells as detected by SAβ-Gal staining were present at E10.5–E14.5, whereas SAβ-Gal activity disappeared after E15.5 (Fig. 1a, b). Therefore, SAβ-Gal activity could be mainly restricted to mid- but not late embryonic stage. These experimental data have been interpreted as indicating that SAβ-Gal^+^ senescent cells underwent apoptosis and were cleared from tissues at late embryonic stage.^8,9^ However, an alternative explanation could be that a subset of senescent cells gradually lost SAβ-Gal activity but survived in the tissue at late embryonic stage. The in vivo senescent cell fate currently remains unknown and untested, as to date there is no fate mapping study on senescent cells.

**Fig. 1:**
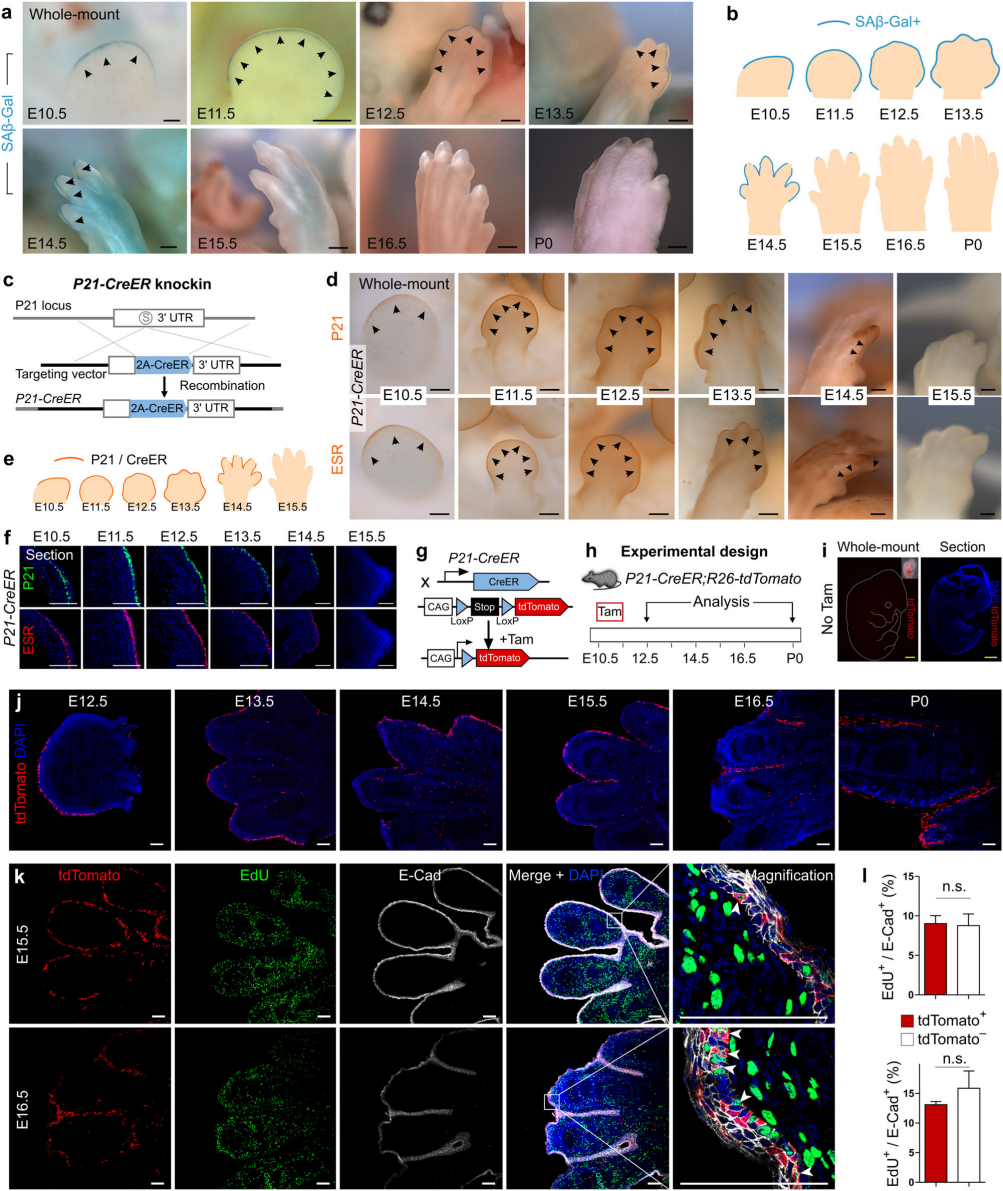
Embryonic senescent cells re-enter cell cycle and contribute to tissues after birth. **a** Whole-mount SAβ-Gal staining on forelimbs of E10.5–P0 mice. Arrowheads indicate SAβ-Gal^+^ cells. **b** Cartoon image showing SAβ-Gal activity pattern. No SAβ-Gal^+^ cell is detected after E15.5. **c** Generation of *P21-CreER* knock-in allele. **d** Whole-mount immunostaining for P21 or ESR on *P21-CreER* embryos. **e** Cartoon image showing expression pattern of P21 and CreER on *P21-CreER* mouse limbs. **f** Immunostaining for P21 and ESR on *P21-CreER* limb sections. **g** Strategy for genetic lineage tracing by tamoxifen (Tam)-mediated Cre-loxP recombination. **h** Schematic figure showing experimental strategy. Tam tamoxifen. **i** Whole-mount and sectional view of tdTomato expression in *P21-CreER;R26-tdTomato* embryo without tamoxifen (No Tam) treatment. **j** Immunostaining for tdTomato on E12.5–P0 mouse limb sections. tdTomato^+^ cells persist after birth. **k** Immunostaining for tdTomato, EdU, and E-cadherin (E-Cad) on E15.5 and E16.5 limb sections. Arrowheads indicate proliferating tdTomato^+^ cells. **l** Quantification of the percentage of proliferating tdTomato^+^ epithelial cells. *n* = 5; n.s., nonsignificant. Scale bars: yellow, 1 mm; black, 200 µm; white, 100 µm. Each figure is representative of five individual samples

P21 is a molecular mediator for embryonic senescence^8,9^ and is highly expressed in SAβ-Gal^+^ senescent cells of AER.^8^ To trace the cell fate of senescent cells during embryogenesis, we generated the *P21-CreER* mouse line by knocking CreER cDNA into the stop codon of P21 (Fig. 1c). 2A self-cleaving peptide sequence was used to allow simultaneous expression of CreER and P21 in P21^+^ cells (Fig. 1c). Immunohistochemistry for P21 or estrogen receptor (ESR, for detection of CreER) in *P21-CreER* mouse forelimbs showed their similar expression patterns to SAβ-Gal activity pattern in embryonic limbs (Fig. 1d, compare to Fig. 1a), suggesting that senescent cells of AER expressed high levels of P21 at mid-stage (e.g., E10.5–E13.5). The CreER expression in embryonic forelimbs at E10.5–E13.5 was largely within AER, recapitulating endogenous P21 expression (Fig. 1d, e). However, the expression of both CreER and P21 was reduced at E14.5 and not detected at E15.5 (Fig. 1d, e). These data demonstrated that CreER was successfully knocked in at the *P21* gene locus (Fig. 1c). We further validated co-expression of P21 and ESR (CreER) in AER by immunostaining (Fig. 1f). Taken together, the above data showed that SAβ-Gal activity and P21 gene expression were highly restricted to senescent cells at AER at mid-embryonic stage, consistent with the previous study.^8^

We next crossed *P21-CreER* with *R26-tdTomato* reporter for genetic lineage tracing of P21^+^ senescent cells during embryogenesis. Tamoxifen pulse treatment leads to translocation of CreER into the nucleus of P21^+^ cells, allowing subsequent Cre-loxP recombination to remove the transcriptional stop region for tdTomato expression (Fig. 1g). We administered tamoxifen at E10.5 or E11.5 to label P21^+^ senescent cells at mid-embryonic stage and then collected tissue samples from E12.5 to birth (P0) for analysis (Fig. 1h). Without tamoxifen treatment, embryos exhibited no detectable tdTomato signal (Fig. 1i), indicating no leakiness of *P21-CreER*. In tamoxifen-treated samples, we could readily detect tdTomato^+^ cells in the AER of developing forelimbs at mid-embryonic stage (Fig. 1j). To confirm that the labeled P21^+^ cells after tamoxifen treatment were indeed senescent cells, we first isolated tdTomato^+^ and tdTomato^−^ cells from E12.5 limbs by fluorescence-activated cell sorting (FACS, Supplementary information, Figure S2a). By co-staining with SAβ-Gal and tdTomato on FACS-isolated cells, we detected SAβ-Gal activity in tdTomato^+^ cells but not in tdTomato^−^ cells (Supplementary information, Figure S2b), demonstrating that P21^+^/tdTomato^+^ cells at E12.5 were senescent cells. The co-expression of SAβ-Gal, P21, and ESR was also confirmed by immunostaining on consecutive sections of *P21-CreER* limbs (Supplementary information, Figure S2c). To further demonstrate that the P21^+^ cells labeled at early stage were senescent cells, we generated *P21-tdTomato* knock-in allele by targeting tdTomato cDNA into the stop codon of P21 with addition of 2A self-cleaving peptide sequence (Supplementary information, Figure S2d). Immunostaining and FACS analysis showed that the tdTomato^+^P21^+^ cells co-express SAβ-Gal at E11.5–E12.5 (Supplementary information, Figure S2e-g). We stained *P21-CreER* tissue sections with additional senescence markers CD44 and HP1γ, and found that CreER^+^ cells in the AER also expressed CD44 and HP1γ (Supplementary information, Figure S3), consistent with previous report.^8,9^ These data showed that the P21^+^ (CreER^+^/tdTomato^+^) cells expressed senescence markers at E11.5–E12.5.

We next followed their cell fate by lineage tracing reporter tdTomato and found that some tdTomato^+^ cells were dispersed between the digits at late embryonic stage (Fig. 1j). Immunostaining for tdTomato and E-cadherin on *P21-CreER;R26-tdTomato* tissue sections showed that tdTomato^+^ cells mainly maintained the epithelial cell fate during embryonic development (Supplementary information, Figure S4). P21^+^ senescent cells labeled at mid-embryonic stage remained in the forelimbs after birth (P0) and exhibited epithelial cell fate (Fig. 1j and Supplementary information, Figure S4). By TUNEL assay, we detected apoptosis of tdTomato^+^ cells in the forelimbs, indicating that a subset of senescent cells died or were removed during development (Supplementary information, Figure S5). By EdU incorporation for analysis of cell proliferation, we found that P21^+^ senescent cells (tdTomato^+^) did not proliferate at E11.5 and E12.5 (Supplementary information, Figure S6), which further confirmed their senescence state. However, a subset of tdTomato^+^ cells began to proliferate at E13.5, albeit at lower rate compared with tdTomato^−^E-Cad^+^ cells (Supplementary information, Figure S6). At E14.5, a similar rate of cell proliferation was detected between tdTomato^+^ and tdTomato^−^ epithelial cells (Supplementary information, Figure S6). At late embryonic stages such as E15.5 and E16.5, ~10% tdTomato^+^ cells were positive for EdU (Fig. 1k, l), indicating their cell cycle re-entry for proliferation. There was no significant difference in EdU incorporation between tdTomato^+^ and tdTomato^−^ epithelial cell populations (Fig. 1l). To further validate this finding, we co-stained *P21-CreER;R26-tdTomato* limb sections with another proliferation marker Ki67 and tdTomato/E-Cad. We found that tdTomato^+^ cells at E11.5 and E12.5 were Ki67^−^, and a subset of tdTomato^+^ cells at late embryonic stages expressed Ki67 (Supplementary information, Figure S7). To quantify the percentage of the remaining tdTomato^+^ cells that had ever entered cell cycle from early to late embryonic stages, we injected EdU every day from E12.5 to E16.5 and then collected limbs for quantification of EdU incorporation. We found that about half of the remaining tdTomato^+^E-Cad^+^ cells have re-entered cell cycle during development (48.74% ± 8.29%, Supplementary information, Figure S8). Interestingly, these previously labeled senescent cells lost senescent hallmarks SAβ-Gal and P21 at E15.5 and E16.5 (Fig. 1a, d and Supplementary information, Figure S9). Senescent cells re-entering cell cycle was not restricted to AER, as we also detected proliferation of previously labeled senescent cells in the endolymphatic sac of inner ear and mesonephros at later embryonic stages (Supplementary information, Figure S10). Taken together, our data provided the first in vivo genetic evidence that previously labeled senescent cells remained in the tissue at late embryonic stage or after birth, and a subset of these post-senescent cells gradually lost senescence markers and proliferated at a comparable rate to their neighbors, the non-senescent epithelial cells.

This work is the first genetic lineage tracing study attempting to dissect in vivo fate of senescent cells. Some senescent cells lost their hallmarks SAβ-Gal and P21, and re-entered cell cycle in vivo during embryogenesis. Rather than being cleared from tissues,^8,9^ a subset of senescent cells proliferated and survived in the tissue after birth. This cell plasticity indicated that senescence could be a transient form of cell cycle arrest in vivo, and this state might not be hard-wired and irreversible in some conditions.^12^ The cell cycle re-entry and loss of the key senescence markers such as SAβ-Gal and P21 indicated that senescence could be readjusted to escape from cell death and clearance during development. Nevertheless, whether adult senescent cells could re-enter cell cycle during aging, cancer, or other diseases remains largely unknown. Recent study indicates that previously labeled senescent cells escape from the arrested condition and re-enter the cell cycle to gain much aggressive tumor growth property.^12^ Genetic targeting of these post-senescent cells in vivo would provide new mechanistic insights into the senescent cell plasticity and development of the associated diseases. It would be a potentially important direction for exploring the intrinsic features of the senescent cells that could be programmed to change their cell fate in future studies. It is also interesting to understand what factors determine the cell cycle re-entry of some senescent cells. It would also be possible that rather than being cleared from aged or diseased tissues for improvement of function,^13–15^ senescent cells might be coached to change their cell fate and function for treatment of aging- or cancer-related diseases in the future.

## Electronic supplementary material

Supplementary information

## References

[1] Hayflick L (1965). Exp. Cell Res..

[2] Childs BG, Durik M, Baker DJ, van Deursen JM (2015). Nat. Med..

[3] He S, Sharpless NE (2017). Cell.

[4] Kang TW (2011). Nature.

[5] Demaria M (2014). Dev. Cell.

[6] Mosteiro Lluc, Pantoja Cristina, Alcazar Noelia, Marión Rosa M., Chondronasiou Dafni, Rovira Miguel, Fernandez-Marcos Pablo J., Muñoz-Martin Maribel, Blanco-Aparicio Carmen, Pastor Joaquin, Gómez-López Gonzalo, De Martino Alba, Blasco Maria A., Abad María, Serrano Manuel (2016). Tissue damage and senescence provide critical signals for cellular reprogramming in vivo. Science.

[7] Baker DJ (2016). Nature.

[8] Storer M (2013). Cell.

[9] Munoz-Espin D (2013). Cell.

[10] Rajagopalan S, Long EO (2012). Proc. Natl Acad. Sci. USA.

[11] Chuprin A (2013). Genes Dev..

[12] Milanovic M (2018). Nature.

[13] Baker DJ (2011). Nature.

[14] Childs BG (2016). Science.

[15] Baar MP (2017). Cell.

