# Supplementary material for: Embryonic senescent cells re-enter cell cycle and contribute to tissues after birth
Source: Cell Res. 2018 Jun 5;28(7):775–8. doi: 10.1038/s41422-018-0050-6 (PMC6028486; doi:10.1038/s41422-018-0050-6)
Supplement: Supplementary file 1 — Supplementary information [file 41422_2018_50_MOESM1_ESM.pdf]

Supplementary Information for:

Embryonic senescent cells re-enter cell cycle and contribute to tissues after birth

Yi Li[1,2], Huan Zhao[1,2], Xiuzhen Huang[1,2], Juan Tang[1,2], Shaohua Zhang[1], Yan Li[1,2], Xiuxiu Liu[1], Lingjuan He[1,2], Zhengyu Ju[3], Kathy O. Lui[4], Bin Zhou[1,2,3,5]

[1]State Key Laboratory of Cell Biology, CAS Center for Excellence in Molecular Cell Science, Institute of Biochemistry and Cell Biology, Shanghai Institutes for Biological Sciences, University of Chinese Academy of Sciences, Chinese Academy of Sciences, Shanghai 200031, China. [2]Key Laboratory of Nutrition and Metabolism, Institute for Nutritional Sciences, Shanghai Institutes for Biological Sciences, University of Chinese Academy of Sciences, Chinese Academy of Sciences, Shanghai, 200031, China. [3]Department of Chemical Pathology; Li Ka Shing Institute of Health Sciences, The Chinese University of Hong Kong, Prince of Wales Hospital, Shatin, Hong Kong SAR, 999077, China. [5]Key Laboratory of Regenerative Medicine of Ministry of Education, Institute of Aging and Regenerative Medicine, Jinan University, Guangzhou, China. [3]School of Life Science and Technology, ShanghaiTech University, Shanghai, 201210, China.

Materials and Methods

Experimental mice

All mice used in our experiments were C57BL/6J background. All mouse operations were done according to the guidelines of the Institutional Animal Care and Use Committee (IACUS) at the Institute for Nutritional Sciences and Institutes for Biochemistry and Cell Biology, Shanghai institutes for Biological Sciences, Chinese Academy of Sciences. Detection of vaginal plug in the morning was designated as E0.5. Tamoxifen (Sigma: T5648-5G) treated by oral gavage at the indicated times (0.05-0.10 mg/g). *R26-tdTomato* mouse line was reported previously[1]. The *P21-CreER* mouse line was generated by homologous recombination using CRISPR/Cas9-mediated genome editing technology. cDNA encoding $CreER^{T2}$ was knocked into the transcriptional stop codon of P21, and a 2A self-cleaving peptides was included before the cDNA to allow simultaneous expression of CreER and P21. We crossed *P21-CreER* with *R26-tdTomato* to perform genetic lineage tracing. *P21-tdTomato* mouse line was generated by conventional homologous recombination in embryonic stem cells (ES). cDNA encoding tdTomato was inserted into the transcriptional stop codon of *P21* gene, and a 2A self-cleaving peptides was included to link P21 and tdTomato to allow expression of both genes. Shanghai Model Organisms Co., Ltd. generate new knockin mouse lines in this study.

Genomic PCR

Genomic DNA was extracted from embryonic yolk sac or mouse tail. Tissues were incubated in proteinase K overnight at 55 °C, followed by centrifugation at 13000rpm for 8min to obtain supernatant with genomic DNA. The supernatant was added equal volume isopropanol to precipitate DNA and washed in 70% ethanol. These steps need centrifugation at 13000rpm for 3min to obtain precipitate with genomic DNA. All DNA was genotyped with specific primers that distinguished the knock-in allele from the wild-type allele. For *R26-tdTomato* line, primers 5'-AAGGGAGCTGCAGTGGAGTA-3' and 5'-CCGAAAATCTGTGGGAAGTC-3' were used to detect the wild type allele. Primers 5'-GGCATTAAAGCAGCGTATCC-3', and 5'-CTGTTCCTGTACGGCATG-3' were used to detect *R26-tdTomato* inserted allele. For *P21-CreER* line, primers 5'-TCAGCAGATGAGGGCACTTACTC-3' and 5'-GGACAGAAGCATTTTCCAGGTATG-3' were used to detect inserted allele. Primers 5'-TCAGCAGATGAGGGCACTTACTC-3' and 5'-AAGGAACCAGGCAGGGAATG-3' were used to detect wild type allele. For *P21-tdTomato* line, primers 5'-TGGCTTTTCCCCACGATGAG-3' and 5'-GAACTCTTTGATGACCTCCTCGC-3' were used to detect *P21-tdTomato* inserted allele. Primers 5'-TGGCTTTTCCCCACGATGAG-3' and 5'-

AAGGAACCAGGCAGGGAATG-3’ were used to detect wild type allele. The programs of PCR used 60°C as the annealing temperature and 35 cycles for amplification of genomic DNA.

SAβ-Gal staining

SAβ-Gal staining was performed according to the Senescence β-Galactosidase Staining Kit (Cell Signaling Technology, #9860). Embryos and tissue sections were incubated in PBS and then fixed in 1X fixative solution (need to dilute 10X fixative solution to 1X with distilled water) at room temperature for 10-15min. After two times washing in PBS, embryos and tissue sections were treated with X-Gal staining cocktail (adjusted pH to 6.0 using NaOH or HCl) at 37°C for 5-6 hours. X-Gal staining cocktail needs to be prepared by mixing with distilled water, 10X staining solution, 100X solution A and 100X solution B. The staining circumstances need to be in low CO_2 condition. Embryos and tissues sections were washed in PBS to stop the reaction and then fixed in 4% fresh PFA for 1 hour. Pictures were taken using a Zeiss stereo microscope (AxioZoom V16).

Whole-mount P21 and ESR staining

Whole-mount immunohistochemistry was performed as previously described[2]. Embryos were collected in PBS and then fixed in 4% paraformaldehyde (PFA) overnight at 4°C. After three times washing with PBT (PBS with 0.1% Tween20) for 10 min at 4°C, embryos were sequentially dehydrated through 25%, 50%, 75%, 100% methanol for 15 min at room temperature. Next, embryos were incubated with 6% hydrogen peroxide (diluted in methanol) for 2 hours with rocking. Rehydrating embryos through 100%, 75%, 50%, 25% methanol for 15 minutes at room temperature. Incubating embryos with primary antibodies (P21, Abcam, ab107099; ESR, Abcam, ab27595) overnight at 4°C, following by 4 times washing in PBT for 1 hour at 4℃ with rocking. Embryos were then left in PBT overnight with constant rotation. Embryos were incubated with secondary antibodies (Immpress anti-rabbit, Vector lab, MP-7401-50; Immpress anti-rat, Vector lab, MP-744-15) for 2 hours at room temperature, followed by 4 times of washing in PBT for 1 hour at 4°C with constant rotation. Embryos were then left in PBT overnight with constant gentle rotation. In the next day, the ImmPACT DAB kit was used to for whole-mount immunohistochemistry. After signal development, the embryos were fixed in the 4% PFA for 1 hour at room temperature. Images were taken using a Zeiss stereo microscope (AxioZoom V16).

TUNEL assay

Tissue sections were fixed in 4% PFA for 10 min at room temperature, followed by washing in PBS for 15 min. The slides were then incubated in PBST (PBS with 0.1% triton X-100) for 2 min at 4°C. Tissue sections were incubated in TUNEL fluorescein (50 µl enzyme solution dilute in 450 µl label solution, Roche, 11684795910) for 1 hour at room temperature. After sections were washed in PBS, immunofluorescent staining was performed at following steps. Images were acquired on Olympus confocal microscope (FV1200).

EdU staining

Pregnant mice were treated with 10 µg/g EdU (ThermoFisher, A10044) at 2 hours before collected through intraperitoneal injection (for quantification of total proliferated ratio, 10 µg/g EdU was treated everyday from E12.5). Tissue sections were blocked in PBSST (5% normal donkey serum in PBST) for 1 hour at room temperature. Click-iT reaction cocktail (Invitrogen, C10337) need to be prepared in 15 minutes before used. Slides were incubated in Click-iT reaction cocktail for 30 min at room temperature in dark, followed by three times washing in PBS for 10 min at room temperature. Immunofluorescent staining was performed at following steps. Images were acquired on Olympus confocal microscope (FV1200).

Immunofluorescent staining

Immunofluorescent staining was performed as previously described[3]. Embryos or tissues were collected in PBS, and then fixed in 4% PFA for 30-50 minutes. After three washes in PBS, embryos or tissues were dehydrated in 30% sucrose (dissolved in PBS) overnight and embedded in OCT (Sakura). Next, cryosections of 9-10µm in thickness were collected. After air dry for 1-1.5 hour at room temperature, sections were incubated with 30% H_2O_2 for 10 minutes. After two times of washing in PBS for 10 min at room temperature, slides were blocked in blocking buffer (5% donkey serum, 0.1% Triton X-100 in PBS) for 30 min at room temperature, followed by incubated with primary antibodies (P21, Abcam, ab107099; ESR, Abcam, ab27595; tdTomato, Rockland, 600-401-379; Ki67, Thermo Scientific, RM-9106-S0; CD44, eBioscience, 14-0441-82; HP1γ, Cell Signaling, 2619S) at 4°C overnight. In the next day, slides were washed in PBS for 15 min and then incubated with secondary antibodies with dilution of 1:1000 (Immpress anti-rat, Vector Lab, MP-744-15; donkey anti-rabbit 555, Invitrogen, A31572; donkey anti-rabbit 647, Invitrogen, A31573; Immpress anti-rabbit, Vector Lab, MP-7401) for 30 min at room temperature in dark. For P21, Ki67, CD44 and HP1γ staining, we used HRP-conjugated antibodies (Vector Lab) with tyramide signal amplification kit (PerkinElmer) to develop the signal for 3-5 min in dark at room temperature. After three times of washing in PBS for 15 min at room temperature,

tissue sections were mounted with mounting medium containing the nuclear stain DAPI (Vector Lab). Images were acquired on Olympus confocal microscope (FV1200). The obtained images were analyzed by ImageJ software (NIH).

FACS and SAβ-Gal assay

The forelimbs were harvested from E12.5 embryos, and then finely minced and transferred to the digestion mix (RPMI1640, 5% FBS, 1% PSG and 300U Collagenase3). Tissues were incubated with the mix for 20-30 minutes at 37°C with gentle shaking. Tissue mix was centrifuged (1000rpm, 5min) and the precipitate was re-suspended in 2ml pre-warmed 0.05% trypsin/EDTA, followed by incubation at 37°C for 3-5 minutes. The mix was centrifuged (1000rpm, 5min) and the precipitate was re-suspended by 2-3 ml pre-warmed serum-free DMEM medium (with 1 μg/ml type I DNase). Samples are incubated at 37°C for 3-5 minutes again, with addition of 10ml PBS to stop the digestion. Samples were filtered through 70 μm cell strainer. Cells were collected by centrifugation at 1000 rpm for 5 minutes. The precipitated cells were re-suspended with PBS and then stained with violet dye (1:1000) at 4°C for 30 minutes. After washing cells with 500ml PBS, centrifuge cells for 4600 g for 3 minutes and discard supernatant. Cells were subsequently stained with EpCAM-APC antibody (eBioscience, 17-5791-82) at a dilution of 1:200 for 30 min at 4°C. Finally, the cells were re-suspended in 500 μl FACS buffer. The stained cells were analyzed and sorted using a FACS Aria II Flow Cytometer (BD Bioscience), and the flow cytometric data was analyzed by Flowjo software.

The FACS-isolated $tdTomato^{+}$ and $tdTomato^{-}$ cells were stained with SAβ-Gal using SPiDER-βGal kit (SG03, Dojindo). Cells were cultured in the dish at 37°C overnight in a 5% CO_2 incubator. The cultured cells were washed and then 1ml Bafilomycin A1 working solution was added for incubation at 37°C for 1h in a 5% CO_2 incubator to inhibit endogenous β–galactosidase activity. Bafilomycin A1 working solution was discarded and cells were incubated with 1ml SPiDER-βGal working solution for 2 hours at 37°C in a 5% CO_2 incubator. After supernatant removal, cells were washed with culture medium twice for 1 hour. The stained cells were fixed at 4% PFA for 5-10 minutes at room temperature, and then stained with DAPI (1:2000) for 15min at room temperature. Images were acquired on Olympus confocal microscope (FV1200).

Statistical analysis

All data were representative of 5-6 individuals, as indicated in each figure legend, and presented as mean values ± S.D.. Statistical comparisons between different sets were made with analysis of normalization and variance, followed by two-sided unpaired Student's *t*-test for analyzing

differences between two data groups. P value < 0.05 was considered to be statistically significant difference. All mice were randomly assigned to different experimental groups. No statistical method was used to predetermine sample size.

References

1. Madisen, L. et al. A robust and high-throughput Cre reporting and characterization system for the whole mouse brain. *Nat Neurosci* **13**, 133-140 (2010).
2. Tian, X. et al. Subepicardial endothelial cells invade the embryonic ventricle wall to form coronary arteries. *Cell Res* **23**, 1075-1090 (2013).
3. Zhang, H. et al. Endocardium Contributes to Cardiac Fat. *Circ Res* **118**, 254-265 (2016).

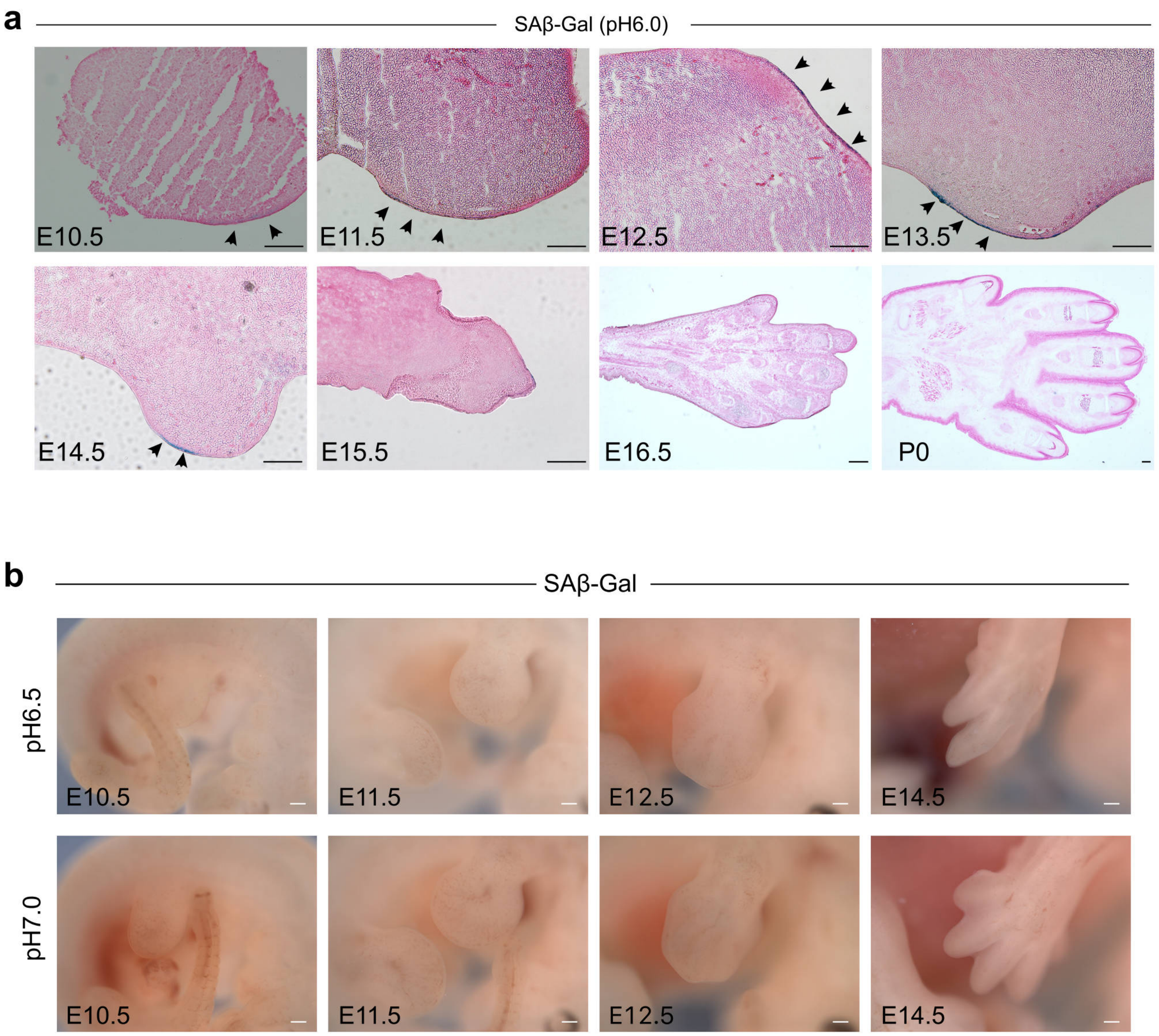

Supplementary information, Figure S1. SAβ-Gal staining on limbs of E10.5-P0 embryos. **(a)** SAβ-Gal staining on tissue sections of E10.5-P0. There is almost no SA-β-Gal⁺ cells in AER at E15.5 and later embryonic stages. **(b)** SAβ-Gal whole-mount staining on embryos at pH6.5 and pH7.0. No visible SAβ-Gal signal was detected. Scale bars, black, 100 μm; white, 200 μm. Each image is representative of 5 individual samples.

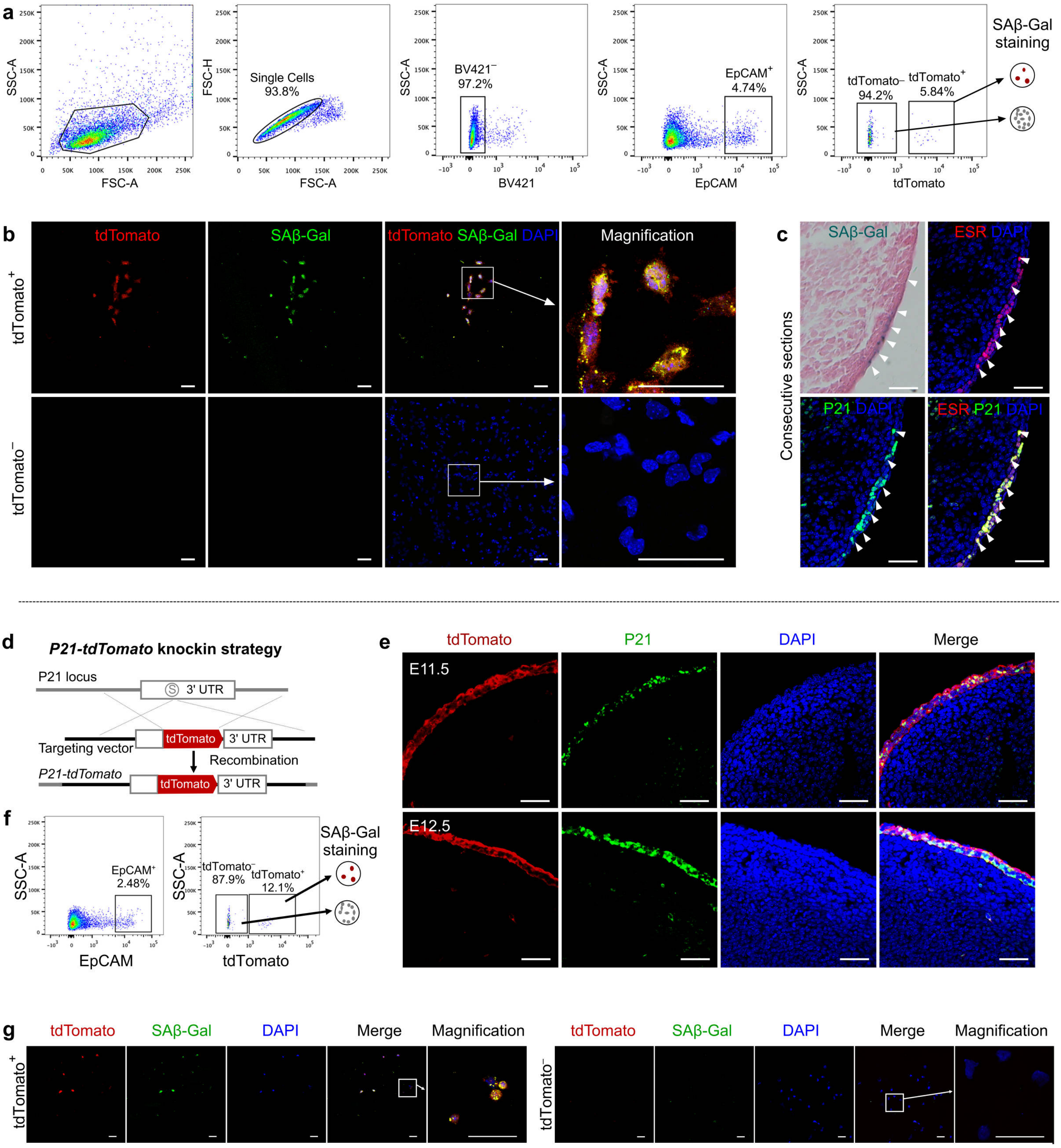

Supplementary information, Figure S2. P21$^+$ cells in the apical ectodermal ridge were SAβ-Gal$^+$ senescent cells. **(a)** FACS isolation of tdTomato$^+$ and tdTomato$^-$ epithelial cells from *P21-CreER;R26-tdTomato* forelimbs at E12.5. **(b)** SAβ-Gal staining on sorted cells shows that tdTomato$^+$ epithelial cells were SAβ-Gal$^+$ and tdTomato$^-$ epithelial cells were SAβ-Gal$^-$. **(c)** Staining for SAβ-Gal, ESR and P21 on consecutive tissue sections. Arrowheads indicate SAβ-Gal$^+$ and ESR$^+$P21$^+$ cells. **(d)** Schematic figure showing knockin of P21-tdTomato allele by homologous recombination. S, stop. **(e)** Immunostaining for tdTomato and P21 on forelimb sections at E11.5 and E12.5. **(f)** FACS isolation of tdTomato$^+$ and tdTomato$^-$ epithelial cells from *P21-tdTomato* limbs. **(g)** SAβ-Gal staining on sorted tdTomato$^+$ and tdTomato$^-$ cells. Scale bars, 50 μm. Each figure is representative of 5 individual samples.

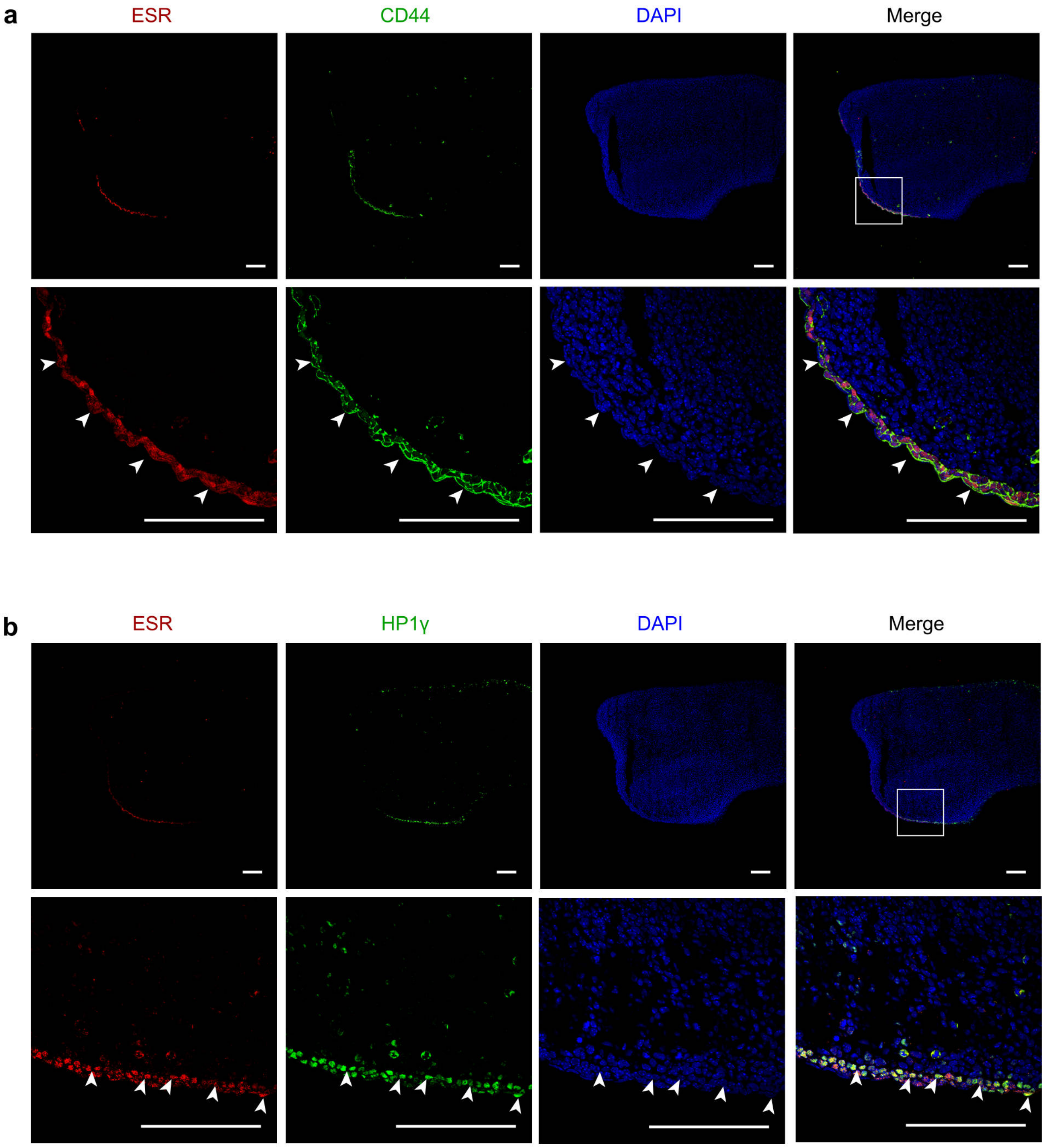

Supplementary information, Figure S3. P21⁺ cells express other senescence cell markers CD44 and HP1γ in the apical ectodermal ridge. **(a)** Immunostaining for ESR (as a surrogate for P21 and CreER) and CD44 on E12.5 limb section*s*. Arrowheads indicate $P21^{+}CD44^{+}$ cells. **(b)** Immunostaining for ESR and another senescence cell marker HP1γ on E12.5 limb sections. Arrowheads indicate $P21^{+}HP1γ^{+}$ cells. Scale bars, 100 μm. Each figure is representative of 5 individual samples.

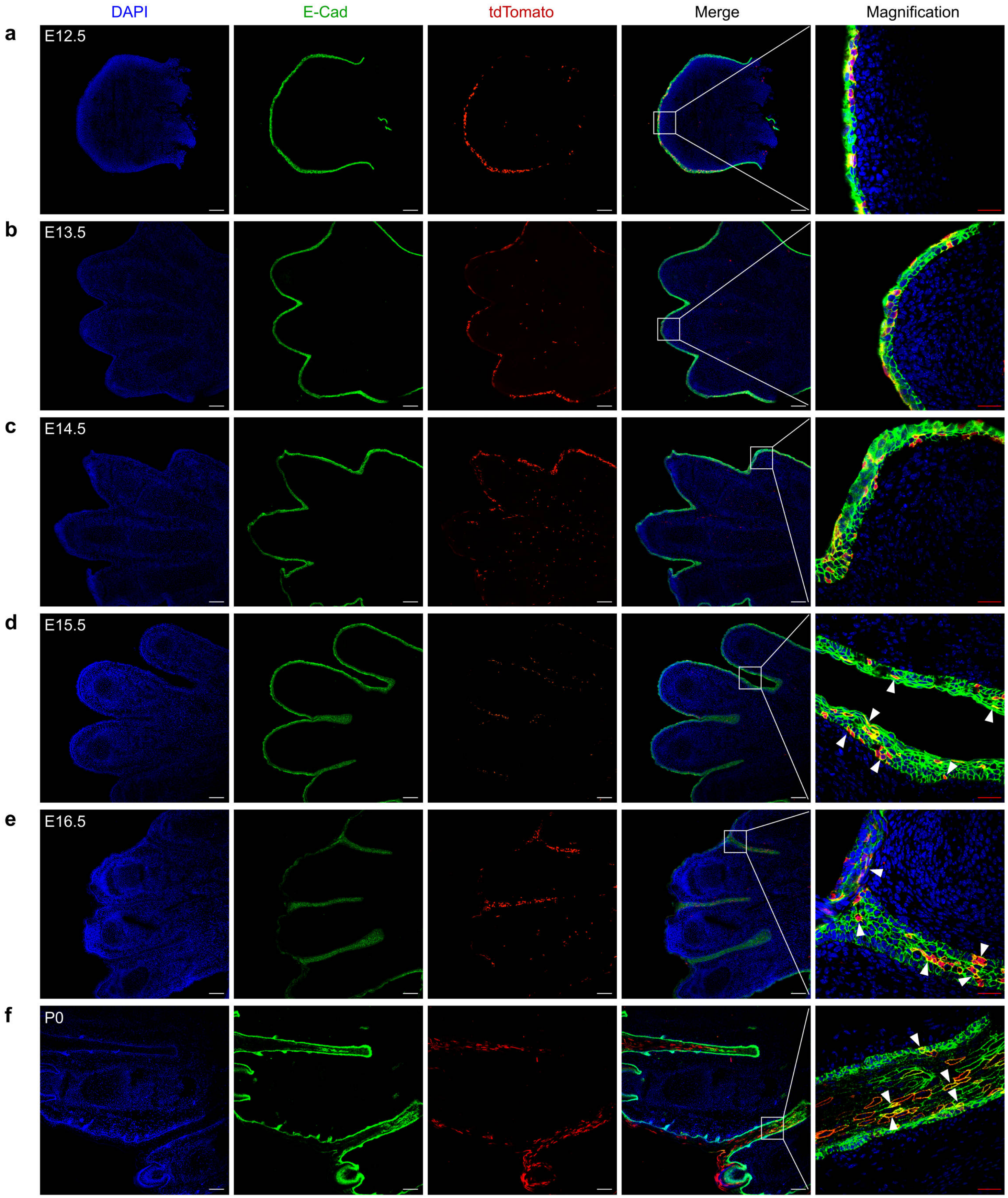

Supplementary information, Figure S4. A subset of senescent cells remain in the limbs at late embryonic stage and after birth. **(a-f)** Immunostaining for E-Cad and tdTomato on sections of E12.5-P0 limbs. *P21-CreER;R26-tdTomato* mice were treated with tamoxifen at E10.5/ 11.5, and limbs were collected at E12.5-P0 for analysis. A subset of *P21-CreER* labeled cells (arrowheads) remain in the limb at late embryonic stage and after birth. Signals of blue and red channels are also shown in Figure 1j, and are co-localized with E-cad here. Scale bars, white, 100 μm; red, 25 μm. Each figure is representative of 5 individual samples.

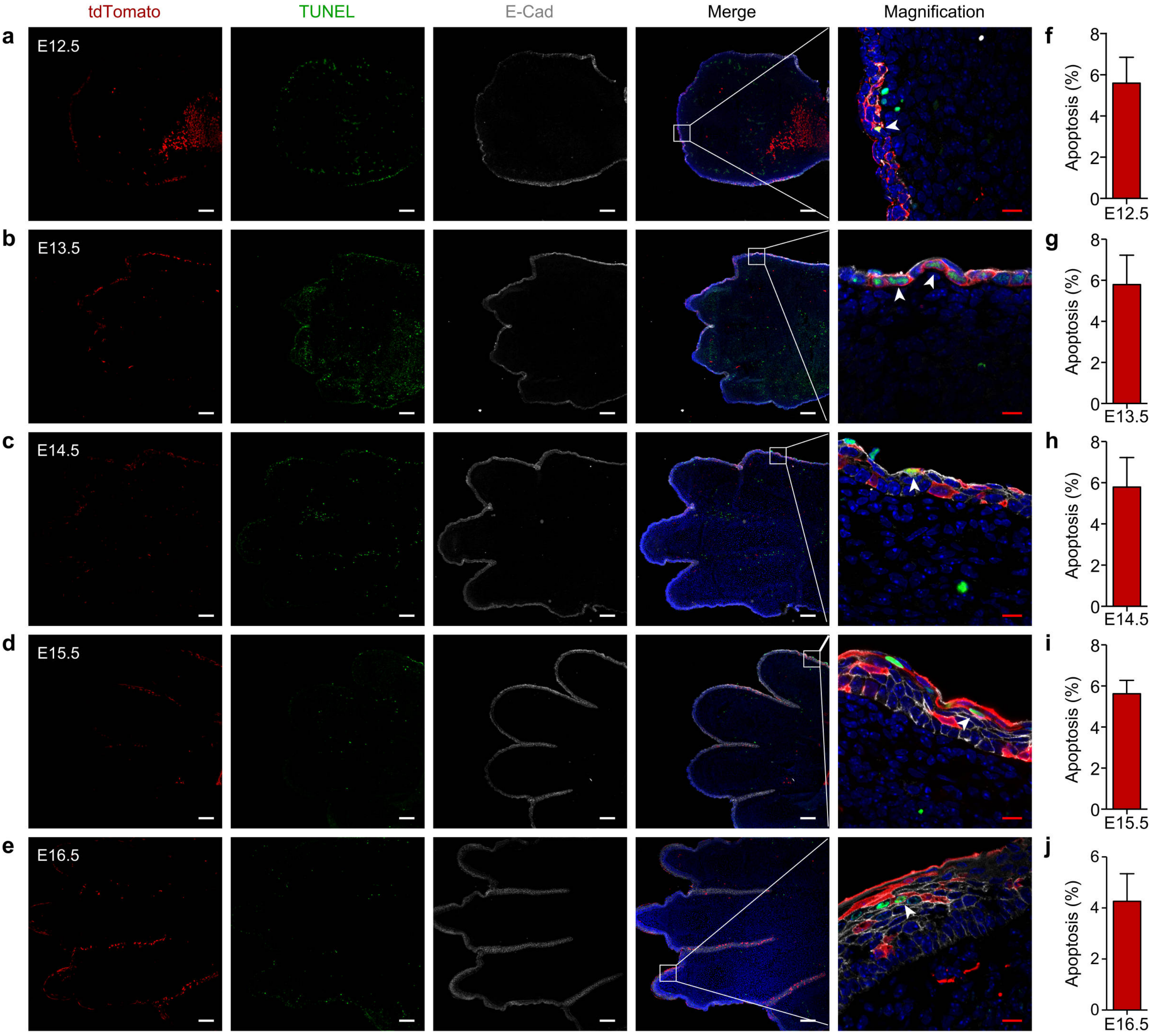

Supplementary information, Figure S5. Senescent cells undergo apoptosis during development. **(a-e)** Immunostaining for TUNEL, E-Cad and tdTomato on limb sections of E12.5-E16.5 embryos. Arrowheads indicate TUNEL$^+$ senescent epithelial cells. **(f-j)** Quantification of the percentage of apoptosis tdTomato$^+$ epithelial cells. n = 5. Scale bars, white 100 μm; red 10 μm. Each figure is representative of 5 individual samples.

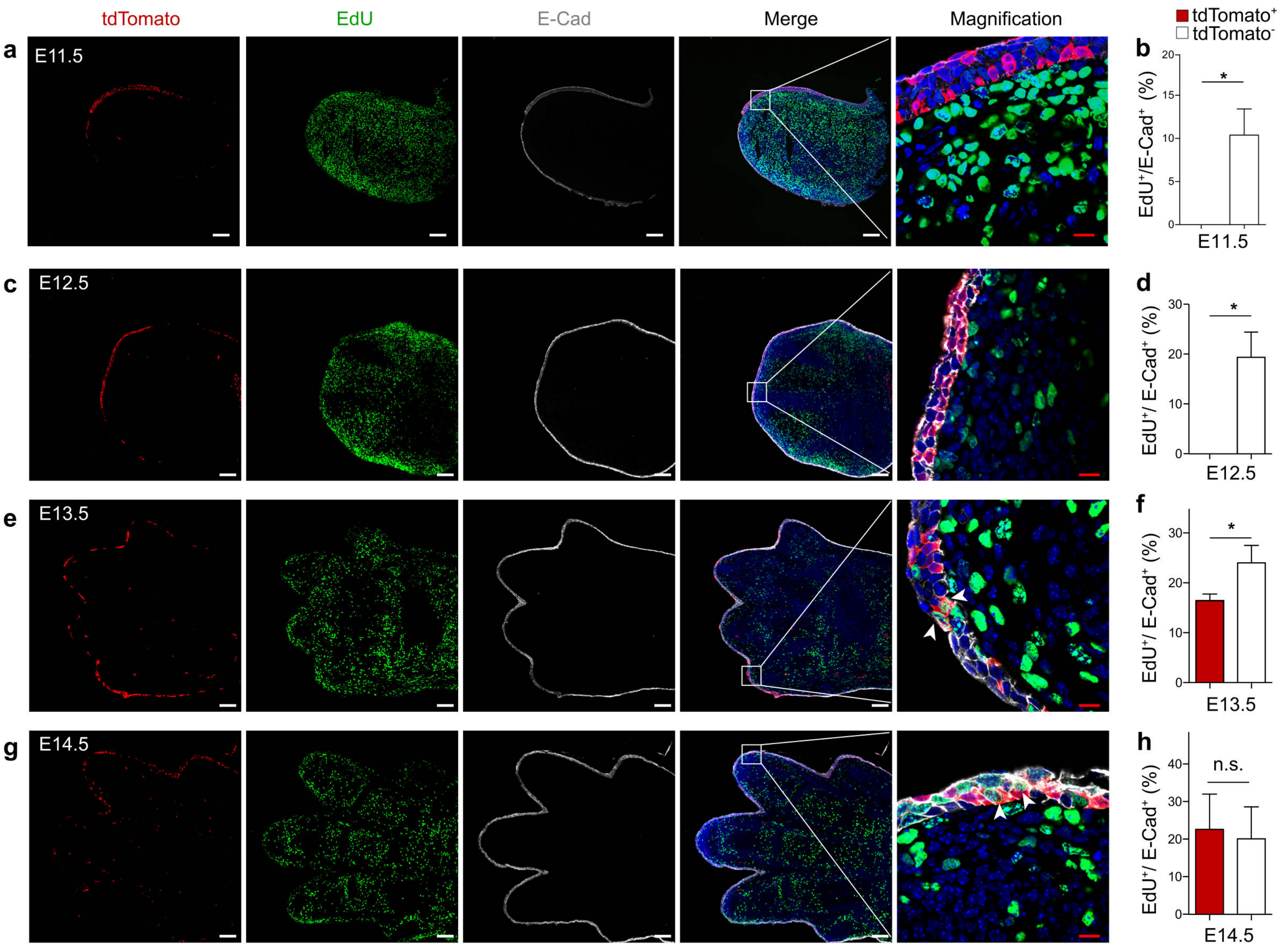

Supplementary information, Figure S6. Descendants of senescent cells entered cell cycle at later embryonic stages. **(a,c,e,g)** Immunostaining for tdTomato, EdU and E-Cad on limb sections of E11.5-E14.5 *P21-CreER;R26-tdTomato* embryos. Mice were treated with EdU 2 hours before sacrifice. $EdU^+tdTomato^+$ cells are not detected at E11.5 and E12.5. Arrowheads indicate EdU^+ $tdTomato^+$ cells detected in the epithelial cell layer at E13.5 and E14.5. **(b,d,f,h)** Quantification of the percentage of $EdU^+tdTomato^+$ cells among $tdTomato^+$ epithelial cells. **P* < 0.05; n.s., non-significant; n = 5. Scale bars, white, 100 μm; red, 10 μm.

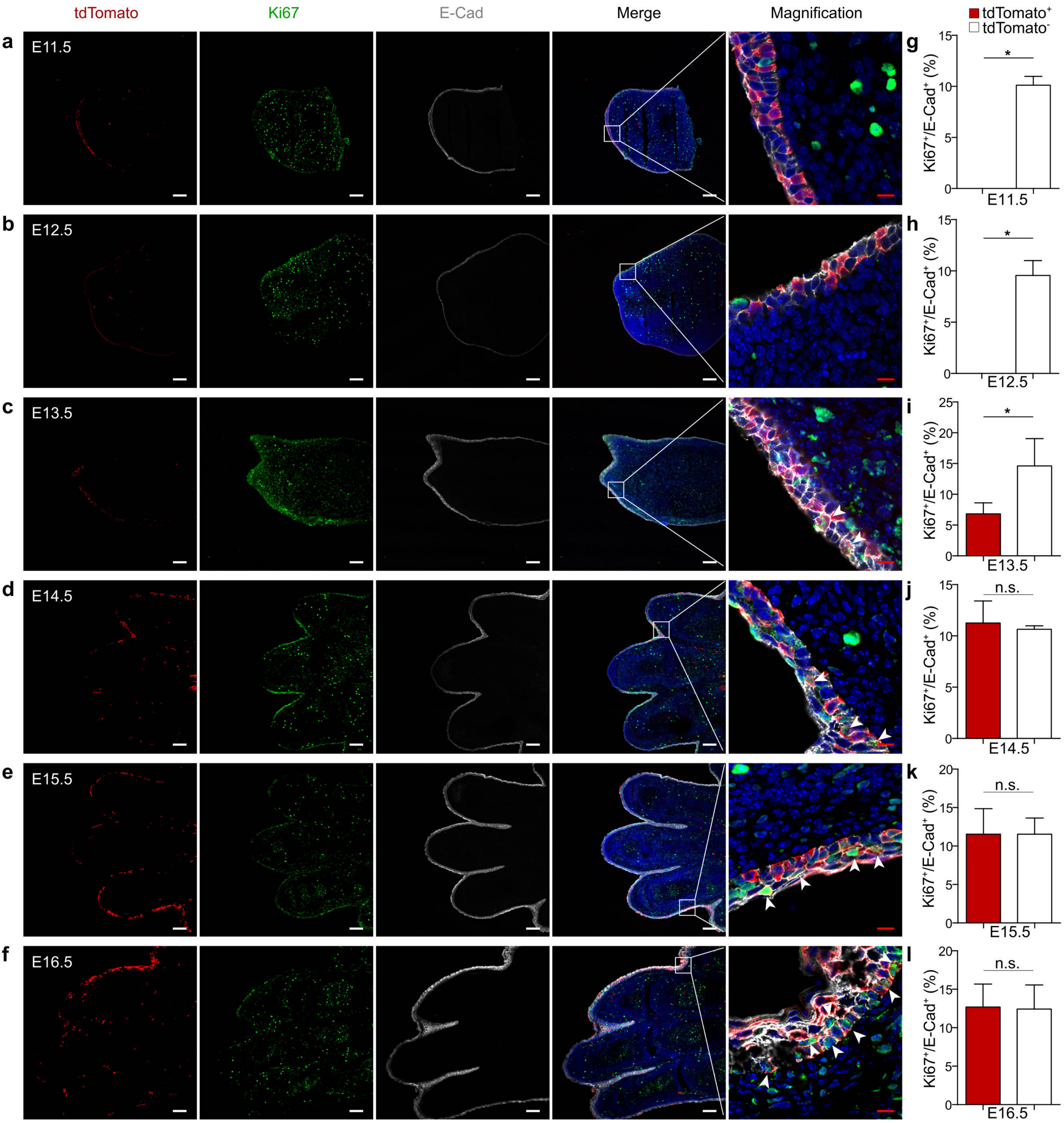

Supplementary information, Figure S7. Descendants of senescent cells express Ki67 at later embryonic stages. **(a-f)** Immunostaining for tdTomato, Ki67 and E-Cad on limb sections of E11.5-E16.5 *P21-CreER;R26-tdTomato* embryos. $Ki67^{+}tdTomato^{+}$ cells are not detected at E11.5 and E12.5; and could be detected at later stages. Arrowheads indicate $Ki67^{+}$ proliferating $tdTomato^{+}$ cells. **(g-l)** Quantification of the percentage of proliferating $tdTomato^{+}$ epithelial cells. n = 5; **P* < 0.05; n.s., non-significant. Scale bars, white, 100 μm; red, 10 μm. Each figure is representative of 5 individual samples.

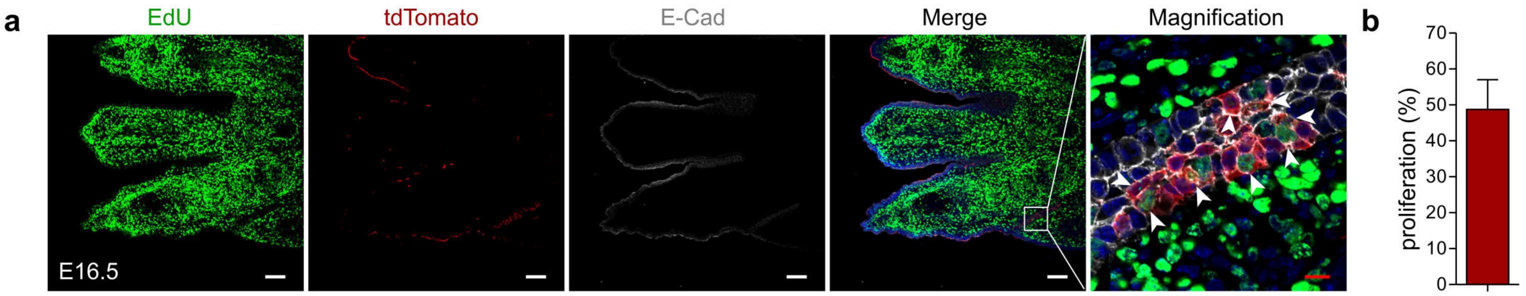

Supplementary information, Figure S8. About half of existing tdTomato+ cells have entered cell cycle in the embryos. **(a)** Immunostaining for tdTomato, EdU and E-Cad on limb sections of E16.5 *P21-CreER;R26-tdTomato* embryos. Mice were treated with tamoxifen at E10.5-E11.5 and then were treated with 10 μg/g EdU everyday from E12.5. **(b)** Quantification of the percentage of proliferating tdTomato+ epithelial cells. n = 5. Scale bars, white, 100 μm; red, 10 μm. Each figure is representative of 5 individual biological samples.

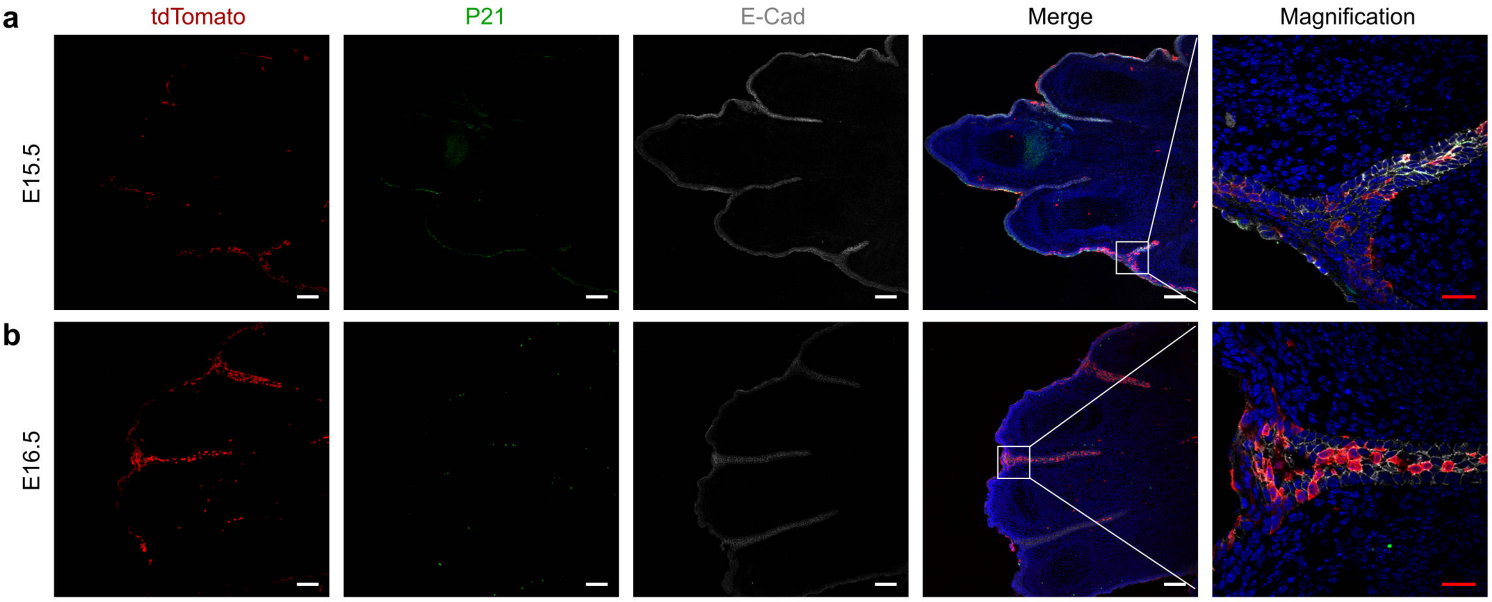

Supplementary information, Figure S9. *P21-CreER* labeled senescent cells lost senescence hallmark of P21 at later embryonic stages. **(a,b)** Immunostaining for tdTomato, P21 and E-Cad on limb sections of E15.5 and E16.5 *P21-CreER;R26-tdTomato* embryos. Tamoxifen was induced at E10.5/11/5. The tdTomato^{+} descendants of senescent cells do not express P21 at these stages. Scale bars, white, 100 μm, red, 25 μm.

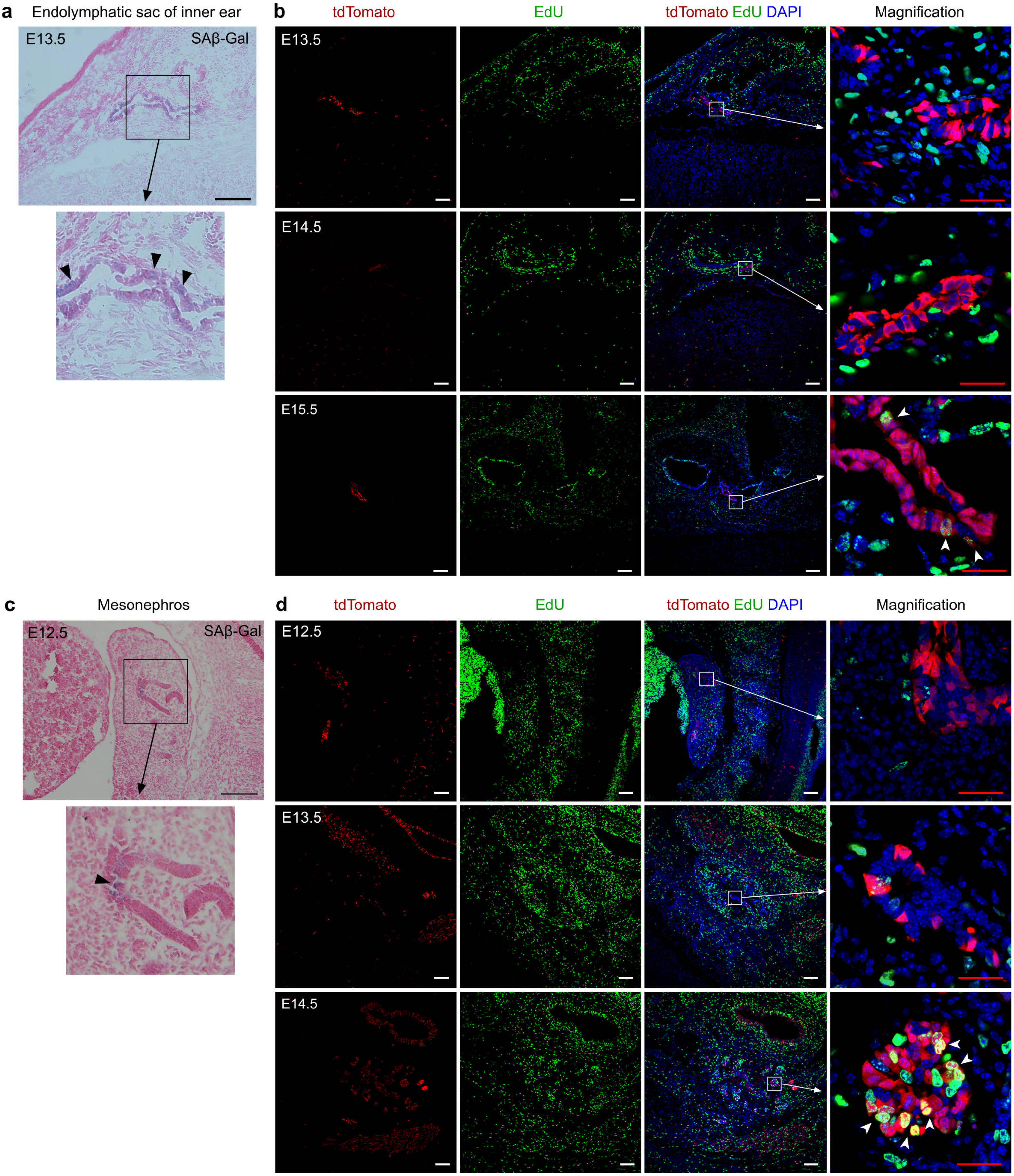

Supplementary information, Figure 10. Analysis of cell senescence and P21$^+$ cell fate in the inner ear and mesonephros. **(a,c)** SAβ-Gal staining of tissue sections from inner ear and mesonephros of E12.5-E13.5 embryos. **(b,d)** Immunostaining for tdTomato and EdU on tissue sections. In the endolymphatic sac of inner ear, tdTomato$^+$ cells do not proliferate at E13.5 (tdTomato$^+$EdU$^-$). A subset of tdTomato$^+$ cells proliferate at E15.5 (EdU$^+$tdTomato$^+$, arrowheads). In mesonephros, tdTomato$^+$ cells do not proliferate at E12.5 (tdTomato$^+$EdU$^-$). A subset of tdTomato$^+$ cells are proliferating at E14.5 (EdU$^+$tdTomato$^+$, arrowheads). Each figure is representative of 5 individual biological samples. Scale bars, white, 100 μm; red, 50 μm.
